# A Man with Severe, Left Lower Quadrant Abdominal Pain

**DOI:** 10.5811/cpcem.1601

**Published:** 2023-08-08

**Authors:** Ta-Jen Wen, Yu-Te Su, Hung-Yen Ke, Kuan-Cheng Lai

**Affiliations:** * Tri-Service General Hospital, National Defense Medical Center, Department of Emergency Medicine, Taipei, Taiwan; † Tri-Service General Hospital, National Defense Medical Center, Division of Cardiovascular Surgery, Department of Surgery, Taipei, Taiwan; ‡ Chiayi Branch, Taichung Veterans General Hospital, Department of Emergency Medicine, Chiayi, Taiwan

**Keywords:** Common iliac artery rupture, diagnostic ultrasound, POCUS

## Abstract

**Case presentation:**

An 84-year-old man presented to the emergency department with sudden, left lower quadrant cramping pain. Because critical hypotension was noted, point-of-care ultrasonography (POCUS) was performed immediately. The study revealed a pulsatile flow extravasating from the left common iliac artery into the left psoas muscle with hypoechoic para-aortic fluid collection.

**Discussion:**

Common iliac artery rupture is rare and has nonspecific clinical presentations. A quick disposition can be made with a combination of clinical manifestations and POCUS results.

## CASE PRESENTATION

An 84-year-old man with a history of hypertension and peripheral arterial occlusive disease presented to the emergency department (ED) with sudden onset of left lower quadrant (LLQ) cramping abdominal pain. The patient was in stable condition with blood pressure 129/61 millimeters of mercury (mm Hg), heart rate 78 beats per minute, and 99% oxygen saturation on room air without respiratory distress on arrival to the ED. Physical examination disclosed mild tenderness over LLQ of abdomen. There was a sudden drop in blood pressure to 66/41 mm Hg with altered consciousness with a Glasgow Coma Score of three (eyes 1, verbal 1, motor 1), while waiting for lab results.

**Image 1:**
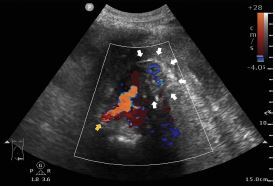
Point-of-care ultrasound in transverse view over left lower quadrant of abdomen demonstrating an extravasating flow from left common iliac artery (yellow arrow) into left psoas muscle (white arrows) under color Doppler display with hypoechoic para-arterial fluid collection.

**Image 2:**
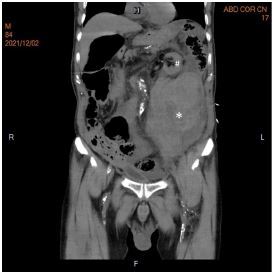
Computed tomography of the abdomen and pelvis, in coronal view, demonstrating a large retroperitoneal hematoma formation (*) with anterior displacement of left kidney (#).

Point-of-care ultrasound (POCUS) over the LLQ of the abdomen showed a left common iliac artery with a demarcated arterial mural defect (Video 1). Pulsatile flow extravasating from the left common iliac artery into the left psoas muscle with a hypoechoic para-arterial fluid collection was noted under color Doppler display (Image 1 and Video 2). Emergent endotracheal intubation and adequate fluid resuscitation with crystalloids was performed immediately. Non-contrast computed tomography was performed subsequently, which revealed a large retroperitoneal hematoma with anterior displacement of the left kidney and descending colon (Image 2). Emergent percutaneous angioplasty was performed based on the suspicion of the rupture of common iliac artery.

## DISCUSSION

**Image 3:**
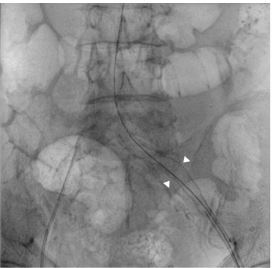
A covered stent (arrowheads) placed in the left common iliac artery by percutaneous transluminal angioplasty.

Common iliac artery rupture is rare and often results from iliac artery aneurysms, dissection, connective tissue disorders (e.g., Marfan, Ehlers-Danlos, and Loeys-Dietz syndromes), atherosclerosis, or even iatrogenesis.^1^ Iliac artery aneurysms generally have nonspecific clinical presentations, including relatively nonspecific severe lower abdomen pain, pulsatile abdominal mass, and bruit.^2^ Due to the rapid progression and hemodynamic instability of arterial rupture a high index of suspicion by the emergency physician is necessary, and POCUS should be considered to make a timely diagnosis.^3^ Sonography may reveal a sharply demarcated aortic mural defect, hypoechoic para-aortic fluid collection, or a heterogeneous collection within a retroperitoneal space. With a combination of clinical manifestations and POCUS results a quick disposition could be made. Percutaneous angioplasty with covered stent (Image 3) in a timely fashion is the main treatment for this emergent condition.^4^

**CPC-EM Capsule**

What do we already know about this clinical entity?

*Common iliac artery rupture is a rare but serious condition often associated with aneurysms, dissection, or connective tissue disorders.*

What is the major impact of the image(s)?

*The images obtained through point-of-care ultrasonography (POCUS) help in timely diagnosis and guide emergent percutaneous angioplasty, potentially preventing fatal outcomes.*

How might this improve emergency medicine practice?

*Increased awareness of this condition and liberal use of POCUS can lead to quicker diagnosis and prompt intervention, improving patient outcomes in cases of common iliac artery rupture.*

## Supplementary Information

Video 1 Point-of-care ultrasound over the left lower quadrant of the abdomen showed a left common iliac artery with demarcated arterial mural defect.

Video 2 Point-of-care ultrasound in transverse view over left lower quadrant of abdomen demonstrating a pulsatile extravasating flow from the left common iliac artery with hypoechoic para-arterial fluid collection.

## References

[1] Altobelli E, Rapacchietta L, Profeta VF (2018). Risk factors for abdominal aortic aneurysm in population-based studies: a systematic review and meta-analysis. Int J Environ Res Public Health.

[2] Azhar B, Patel SR, Holt PJ (2014). Misdiagnosis of ruptured abdominal aortic aneurysm: systematic review and meta-analysis. J Endovasc Ther.

[3] Rubano E, Mehta N, Caputo W (2013). Systematic review: emergency department bedside ultrasonography for diagnosing suspected abdominal aortic aneurysm. Acad Emerg Med.

[4] Wanhainen A, Verzini F, Van Herzeele I (2019). Editor’s Choice - European Society for Vascular Surgery (ESVS) 2019 Clinical Practice Guidelines on the Management of Abdominal Aorto-iliac Artery Aneurysms. Eur J Vasc Endovasc Surg.

